# Beneficial Root Endophytic Fungi Increase Growth and Quality Parameters of Sweet Basil in Heavy Metal Contaminated Soil

**DOI:** 10.3389/fpls.2018.01726

**Published:** 2018-11-27

**Authors:** Mayada Sabra, Amal Aboulnasr, Philipp Franken, Erica Perreca, Louwrance Peter Wright, Iris Camehl

**Affiliations:** ^1^Leibniz-Institute of Vegetable and Ornamental Crops (IGZ), Großbeeren, Germany; ^2^Agriculture Botany Department, Faculty of Agriculture, Alexandria University, Alexandria, Egypt; ^3^Max Planck Institute for Chemical Ecology, Jena, Germany

**Keywords:** arbuscular mycorrhizal fungi, *Rhizophagus irregularis*, *Ocimum basilicum*, *Serendipita indica*, *Piriformospora indica*, heavy metal, soil pollution

## Abstract

How interactions between plants, the rhizosphere, and contaminated soil affect environmental sustainability is still under research. We tested the effects of two root endophytic fungi, the arbuscular mycorrhiza fungus (AMF) *Rhizophagus irregularis* and the beneficial endophyte *Serendipita indica*, on sweet basil (*Ocimum basilicum*) growing on soil contaminated with lead and copper in a pot experiment under defined greenhouse conditions. Both fungi caused an increase in shoot and root dry weight of sweet basil plants under all conditions and decreased the amount of lead in shoots. The amount of copper was reduced by *S. indica*, while the AM fungus showed this effect only when the soil is contaminated with both copper and lead. Furthermore the AMF, but not the endophyte *S. indica* caused a strong increase on the concentrations of the essential oils linalool and eucalyptol even on sweet basil growing on contaminated soils. Hence, cultivating sweet basil in combination with beneficial fungi in case of difficult environmental conditions could be of interest for industry located in countries with widespread land pollution, because quantity and quality of plants are increased while the amount of heavy metals is generally reduced.

## Introduction

As a result of urbanization and industrialization, heavy metal pollution of the soil is a serious problem. It is especially important that soil pollution is kept at a low level that is below the mandatory toxicity limits, when cultivating food crops, or medicinal plants such as sweet basil. In particular, lead (Pb) pollution is a global problem due to its use as an additive in fertilizers and gasoline since the 1920s. On the one hand, there is a trend toward the protection of the environment from lead. But on the other hand, most sewage sludge and other fertilizers used in agriculture are still loaded with lead. Plant uptake is the most important gateway of heavy metals into the food chain and thus into the human body (Guerra et al., 2012). Although lead accumulated in soil is not readily absorbed by plant roots, studies show that it inhibits plant metabolic processes such as root growth, photosynthesis, and water uptake (Sharma and Dubey, 2005; Fahr et al., 2013). Thus, lead is still one of the main sources of environmental pollution and impacts agroecosystems, the environment, and, consequently, human health.

In contrast to lead, copper (Cu) is not easily accumulated in the food chain and thus the toxicity to humans and other mammals is relatively low. Additionally, copper is an essential element to plants and is needed for seed production, lignin synthesis, and the biosynthesis of different enzymes. However its concentrations are often elevated in the soil due to the widespread use of copper-containing fungicides, pesticides, and herbicides, especially in soils used for citrus and grapevine production (He et al., 2005). Plants are in general very sensitive to even slight increases in tissue copper contents. At high doses, copper inhibits root morphology, enhances reactive oxygen species (ROS) production in the plant, and induces the expression levels of genes related to an oxidative stress response (Rastogi et al., 2017).

The annual herb sweet basil (*Ocimum basilicum*, L.) is one of the best studied plants among the 150 *Ocimum* species. It belongs to the family of the Lamiaceae and is of high economic interest. It is mainly cultivated as a spice for the food industry, but also to use its essential oil in cosmetics, pharmaceuticals, and pesticides. The essential oil of sweet basil mostly consists of monoterpenes, sesquiterpenes, and phenylpropanoids with the monoterpene linalool and the phenylpropene methyl chavicol (Estragole) being the major compounds (Lee et al., 2005). Some aromatic plants are exclusively grown for the production of essential oils and Zheljazkov et al. (2006) showed that aromatic plants can be grown in heavy metal enriched soils without the risk of metal transfer into the oils.

Extensive previous research exists on different varieties and cultivars of sweet basil under the influence of beneficial fungi such as AMF, with respect to the biosynthesis and composition of essential oils, and the generation of biomass and plant fitness (Copetta et al., 2006; Toussaint et al., 2007; Zuccarini and Okurowska, 2008). AMF are particularly important to improve the quality of medicinal plants such as sweet basil. The symbionts can be used to enhance nutrient uptake and alter secondary metabolite content (Harrier, 2001). The AM fungus *Rhizophagus irregularis* (formerly known as *Glomus intraradices* (Krüger et al., 2012)) is widely used in research on AM symbiosis, in particular on how it supports plant fitness (Smith and Read, 2008). Going one step further, Battini et al. (2016) described the impact of dual inoculation with AMF and its associated bacteria and found an improvement of nutraceutical value of sweet basil. Nevertheless, little research has been initiated on the impact of beneficial fungi on sweet basil under heavy metal stress. However many investigations of the responses of other plants to heavy metal stress inoculated with AMF exist and different plant mechanisms, including the role of mycorrhizal fungi for heavy metal tolerance are surveyed by Hall (2002). Ingle et al. (2017) showed that AMF play an important role in the alleviation of cadmium stress in chickpea plants. In sweet basil, Prasad et al. (2011) discovered that *R. irregularis* enhance the metal concentration in shoot tissues grown in soil with a low dose of metals, whereas this effect was reciprocal in soil with a high amount of heavy metals.

In addition, the antioxidative plant defense to heavy metals is widely described and the protective impact of AMF in the case of heavy metal stress is also discussed in the literature (Schützendübel and Polle, 2002; Sytar et al., 2013; Shahid et al., 2017). Similarly, previous research exits on the response of AMF itself to heavy metals as well as its capability to decrease heavy metal stress in other plants. *R. irregularis* is able to grow in media with copper concentrations that are lethal to a majority of other organisms (Tamayo et al., 2014) and the effect of decreasing heavy metals in plants is an important aspect for the application of AMF in sustainable agricultural systems (Schreiner and Bethlenfalvay, 1995).

The mutualistic endophytic fungus *Serendipita indica* (formerly known as *Piriformospora indica*) is also widely used in plant-fungus interaction studies. This soil borne fungus originates from the Indian Thar desert and was initially described in Verma et al. (1998). It colonizes roots of diverse plant species and is known to promote growth and disease resistance in its hosts (Varma et al., 1999; Waller et al., 2005). *S. indica* alters the secondary metabolites of many plants of economic importance and, for example, promotes the uptake of iron and copper in the sugarcane ratoon crop (Varma et al., 2012). A recent study shows that *S. indica* is able to protect *Oryza sativa* from arsenic toxicity (Mohd et al., 2017).

In this study we used the two beneficial fungi, *R. irregularis* and *S. indica*, to investigate their interaction with sweet basil growing in Pb and Cu-contaminated soil. We analyzed sweet basil performance concerning biomass, P uptake, heavy metal accumulation and essential oil content. Because plants in natural or anthropogenic terrestrial ecosystems are usually not dealing with only one single factor at time, we choose a total factorial design for our study.

## Materials and Methods

This experiment was carried out in the greenhouse at the Leibniz Institute for Vegetables and Ornamental Crops, Erfurt, Germany in 2015.

### Experimental Design

A fully crossed-four factor design was chosen with three heavy metal treatments, two root colonizing fungi and controls. The AM fungus *R. irregularis* and the root-endophytic fungus *S. indica* were used. The treatment combinations were arranged in a randomized block design with 9 replicates.

### Plant Cultivation

The seeds of *O. basilicum* were obtained from the Medicinal and Aromatic plants Department of the Agricultural Research center, Ministry of Agriculture, Cairo, Egypt. Seeds were germinated in specific peat for small plant cultivation (Pikiererde, Einheitserdewerke Werkverband e.V., Sinntal-Altengronau, Germany) at 25: 18°C (day: night) and irrigated three times a week with 65% holding capacity of water. Four weeks after sowing, single seedlings were transferred to pots (20 cm × 30 cm) filled with 2700 mL of substrate (50% peat and 50% autoclaved sand) and fertilized twice per month with 100 mL/plant low phosphorous mineral fertilizer (Flory 2, which contains 1% P, 15% N, 5% P_2_O_2_, 25% K_2_O, and 2% MgO, Germania Care GmbH, Germany).

### Treatments

At transplanting, plants were treated either with *R. irregularis*, *S. indica* or both simultaneously. AM plants received viable propagules of *R. irregularis* (INOQ GmbH, Schnega, Germany) inoculum (sand contained about 220 mycorrhizal units/cm3) at 10% v/v mixed throughout the soil. One mycorrhizal unit is a spore, a hyphae or a mycorrhizal root fragment. Control plants were treated with the same amount of sand without any microorganisms in order to create the same soil conditions. *S. indica* was grown on PDA plates for 2 weeks (Carl Roth, Karlsruhe, Germany). A small hyphal plug was transferred in 500 mL conical flasks with liquid complete media (CM: 50 mL 20x salt solution (120 g NaNO_3_, 10.4 g KCl, 10.4 g MgSO_4_ × 7 H_2_O, and 30 g KH_2_PO_4_ in 1 L), 20 g glucose, 2 g peptone, 1 g yeast extract, 1 g casamine acids and 1 mL microelements (6 g MnCl_2_, 1.5 g H_3_BO_3_, 2.65 g ZnSO_4_ × 7 H_2_O, 750 mg KI, 2.4 mg NaMO_4_ × 2 H_2_O, 130 mg CuSO_4_ × 5 H_2_O dissolved in 1 L) and incubated for 3 weeks in the dark at 24°C. This liquid fungal culture was blended in a kitchen blender to obtain a mixture as homogeneous as possible. Afterward, this liquid suspension of *S. indica* hyphae and spores was used to soak plant roots of sweet basil. Control sweet basil seedling received the same amount of autoclaved (121°C for 20 min) *S. indica* suspension (mock treatment). The application of lead nitrate [Pb(NO_3_)_2_ × 7 H_2_O, Carl Roth, Karlsruhe, Germany] solution and/or copper sulfate (CuSO_4_ × 5 H_2_O, Carl Roth, Karlsruhe, Germany) solution to the potting substrate was conducted either separately for the single heavy metal treatment (400 mg/L) or in combination (200 mg/L each). An amount of 400 mg/L was chosen because higher amounts induced plant toxicity. Plants in the control group remained untreated. One week after transferring the seedlings to the pots and inoculating them with the fungi, the plants were irrigated every day with 100 ml of the stock solution (400 mg/L) containing the appropriate metal ions over a period of 10 days, to avoid a shock for the plants and to gradually achieve 400 mg total heavy metal amount in the soil. Thereafter plants were irrigated with tap water. The factorial design of the experiment resulted in total in 16 treatments (see Table 1), each with 9 replicates.

**Table 1:** Overview of treatments.

		Microorganisms			
		Mock	*S. indica*	AM	*S. indica* and AM
**Heavy metals**	Soil without heavy metals	x	x	x	x
	Pb	x	x	x	x
	Cu	x	x	x	x
	Cu and Pb	x	x	x	x

### Harvest

The plants were harvested 5 weeks after transplanting and inoculating. This time period was chosen to keep the plants from flowering. Shoots and roots were separated and the plant height (cm), the shoot fresh weight (g/plant), and the number of leaves were measured. Aliquots of 300 mg root material were stored in 70% ethanol to evaluate the mycorrhization through microscopy and histology, and 100 mg of root material was frozen in liquid nitrogen to assess *S. indica* colonization with PCR. Shoots and roots dry weights were determined after drying in an oven at 65°C for 3 days. Samples were ground in a high speed rotor mill (Pulverisette 14, Fritsch, Idar-Oberstein, Germany) to measure lead, copper and oil content in shoots and roots and the phosphorus content in the shoots. Since oil values were compared to each other without the need of the absolute values, a potential loss of volatiles during the drying process was acceptable.

### Histology, Microscopy and Detection of *R. irregularis* in Colonized Roots

Root samples were cut into small pieces of (1–2 cm), bleached in 10% KOH for 15 min at 90°C, washed with water, and submerged in 2N HCl for 2 min. Then they were stained with Trypan blue (0.05%) in lactoglycerol for 15 min at 90°C in a water bath and bleached in lactic acid according to Koske and Gemma (1989). Ten pieces were put on slides and observed under a light microscope with 200 times magnification. AM fungal structures (hyphae, arbuscular, and vesicles) were estimated quantitatively according to Biermann and Linderman (1981). For the histological sections, roots, dehydrated by increasing the ethanol concentrations from 70 to 99% and embedded in paraffin, were cut into 6–12 μm sections using a Leica RM2155 microtome (Leica, Wetzlar, Germany) and placed on a microscope slide. Furthermore, the staining method W-3A described by Wacker (2006) was used. The samples were stained with 1% acridine red for 5–15 min and washed 15 min with distilled water to stain the cell walls blue. The second staining was done with 1% acriflavine for 5–15 s and washed with distilled water for 5 s to stain the fungal hyphae yellow. The last staining was done with 2% astra blue for 30–180 s and then washed off completely for 10–20 s with distilled water to stain other parts of the cell wall red. For the staining solutions see: https://www.waldeck-ms.de/de/produktion/farbstoffe-und-farbstoffloesungen/. The samples were examined with an Olympus BX53 light microscope (Olympus, Tokio, Japan).

### Phosphorus Concentration

Phosphorus (P) uptake was analyzed according to a modified method after Goehler and Drews (1978), Ehrenberger (1991), and Ghanem et al. (2014). Dried and pulverized randomly selected samples of shoot tissues (250 mg per sample) were subjected to an acid digestion (adding 2 mL of 65% HNO_3_ directly to the sample with 2 mL of 30% hydrogen peroxide and 5 mL H_2_O placed outside the sample vessel) at 200°C for 15 min in a microwave (μPREP-A microwave system, MLS GmbH, Leutkirch, Germany). The digested solution was diluted to 50 mL with deionized water. To an aliquot of 5 mL of the sample solution, 10 mL of vanadate–molybdate reagent (Sigma-Aldrich, Steinheim, Germany) was added. After 5 min incubation, P concentration was determined at 440 nm using a V-550 spectrophotometer (Jasco GmbH, Grossumstadt, Germany). Results were calculated in mg P × g^-1^ dry weight.

### Determination of Lead (Pb) and Copper (Cu) Concentrations in Shoots and Roots

Dried and finely ground plant material from randomly selected plants (0.5 g) was digested with ten milliliters of concentrated HNO_3_ (ultrapure 65%) by incubating overnight at room temperature and heating for 4 h at 120°C. The temperature was then increased to 140°C until 1 mL of acid remained and samples were filtered after cooling. Pb and Cu concentrations were determined with atomic absorption spectrophotometry (Analyst 400 Perkin Elmer, England) (Jackson, 1967). Results were calculated in mg Pb or Cu, respectively × kg^-1^ dry weight.

### Essential Oil Measurement

Linalool, methyl chavicol, eucalyptol, and eugenol were extracted from 50 mg of dry herb tissue with 600 μL of TBME (tetra butyl methyl ether) containing isobutylbenzene as internal standard (1.3 μg/μL). The extract was prepared by continuous shaking for 3 h, removed and washed with 500 μL of 0.1M (NH_4_)_2_CO_3_, pH 8.0. After centrifuging for 5 min at 17,000 g, the organic upper phase was passed through a Pasteur pipette, filled with 50 mg of anhydrous Na_2_SO_4_ and 50 mg of silica held in place with a plug of glass wool.

The extracted sample was analyzed by gas chromatography - mass spectrometry (GC-MS) to confirm peak identities and quantified with GC using flame ionization detection (FID). GC analysis was carried out with a Zebron ZB-5 capillary column (Phenomenex, 5% phenyl-95% dimethylpolysiloxane film, 30 m long × 0.25 mm id, 0.25 μm film thickness). Split less injection of 1 μl sample was performed using an injector temperature of 220°C. The instrument was programmed with an initial temperature of 40°C for 3 min, which was increased at a rate of 2°C/min up to 80°C. The temperature was then increased at a rate of 5°C/min to 160°C, following by a rate of 60°C/min up to 300°C. Hydrogen was used as carrier gas at a constant flow of 2 mL/min. The MS scan was set at 50 to 350 m/z, starting after a 5 min solvent delay. Ionization was done through an electron impact at the standard 70 eV. The mass selective detector transfer line heater was at 230°C, the quadrupole of the MS at 150°C and the MS source at 230°C. Compounds were identified by the comparison of their GC retention times and mass spectra with those of authentic standards. To quantify the compounds, a five point calibration was obtained with the authentic standard (Bosch et al., 2014).

### RNA Isolation and PCR Analysis

Fresh root material was transferred directly into liquid nitrogen and stored at -80°C for the detection of *S. indica* activity by RT-PCR. Frozen material (100 mg) was ground with a tissue homogenizer (Precellys 24, VWR International GmbH, Darmstadt, Germany) at -12°C. Total RNA was extracted with the RNeasy plant Mini Kit from Qiagen (Qiagen, Hilden, Germany) according to manufacturer’s instructions, including the on-column-DNAse treatment for 30 min. The quality and quantity of the RNA was monitored with the NanoDrop 2000 (VWR International GmbH, Darmstadt, Germany). 500 ng of RNA was transcribed to cDNA in a final volume of 10 μl with the QuantiTect reverse transcription kit from Qiagen (Qiagen, Hilden, Germany) according to manufacturer’s instructions. Synthesized cDNA was used as a template for PCR to detect *S. indica* with *ITS* primers (forward: CAACACATGTGCACGTCGAT; reverse: CCAATGTGCATTCAGAACGA). As control, a fragment of the actin-encoding gene of *O. basilicum* was amplified [forward: AGATTCCTCCAGCAAATCTTTCTC; reverse: CTTTCTGGTGGAACAGCATCAA (Rastogi et al., 2014)].

### Statistical Analysis

**FIGURE 1:**
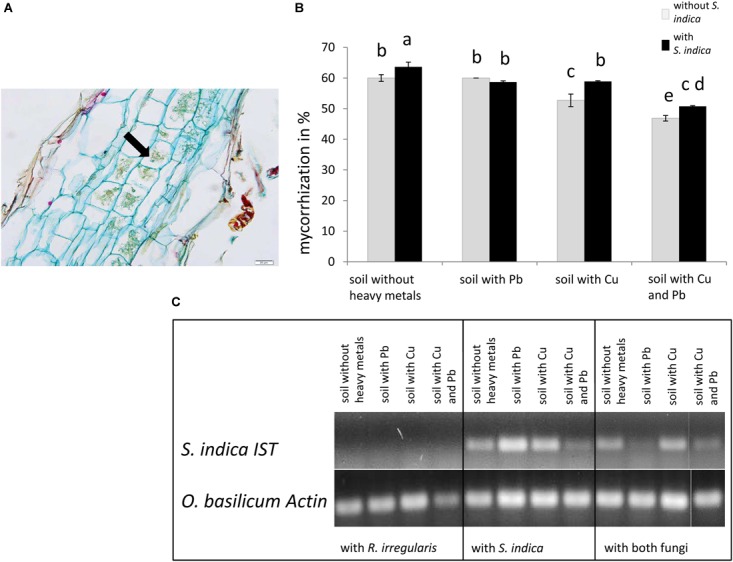
Colonization of sweet basil roots with *Rhizophagus irregularis* and *Serendipita indica* after 5 weeks of inoculation. **(A)** Histological section of a mycorrhizal sweet basil root stained with acridine red, acriflavine and astra blue. The arrow indicates cells with arbuscules. Scale bar: 20 μm. **(B)** Mycorrhizal colonization in %. Gray bars indicate sweet basil plants only inoculated with the AM fungus *R. irregularis* while black bars indicate plants inoculated with both, *R. irregularis* and *S. indica*. Data show means of three individual plants and ten 1 cm root segments per plant with standard error. Bars topped by the same letter do not differ significantly at *p* ≤ 0.05 according to Tukey’s HSD test (see Supplementary Table S1), where all factors do interact, calculating heavy metal treatment as one factor. **(C)** Qualitative detection of *S. indica* in sweet basil roots with PCR, using the *ITS* gene of *S. indica*. Representative samples are shown, which are either inoculated with *R. irregularis* or *S. indica*.

With the program package Statistica (version 12, Tulsa, OK, United States) an analysis of variance (ANOVA) was conducted. Tukey HSD (honestly significant difference) was performed at *p* ≤ 0.05 to detect significant interactions between factors while taking into account the fact that multiple comparisons are necessary due to the experimental design. Lead and copper treatment was considered as single factor (except for Figure 1), respectively and each *S. indica* and *R. irregularis* inoculation was also considered as a single factor, respectively. For detailed information see Supplementary Tables S1–S9. If no significant interaction between all factors was observed, the mean values were compared pairwise at *p* ≤ 0.05 with Student’s *t*-Test. All data are shown as mean values with standard errors.

## Results

### Fungal Colonization

Mycorrhization was detected and quantified in the roots by staining, controls remain untreated and no colonization was observed (Figures 1A,B and data not shown). The highest level of mycorrhization was discovered in plants inoculated with both fungi on soil neither treated with copper nor with lead with 63% AM fungal structures in root pieces (Figure 1B). Only the simultaneous application of copper and lead significantly decreased mycorrhization. When both fungi were present, the mycorrhization decreased significantly in lead contaminated soil compared to normal soil. The lowest mycorrhizal colonization with only 46% was visible when only *R. irregularis* was present and the soil was contaminated with both heavy metals (Figure 1B). In general we found that mycorrhization increased with the presence of *S. indica*, except in lead contaminated soil. Furthermore the mycorrhization intensity decreased when the soil was contaminated with copper but not with lead.

### Growth Parameters

**FIGURE 2:**
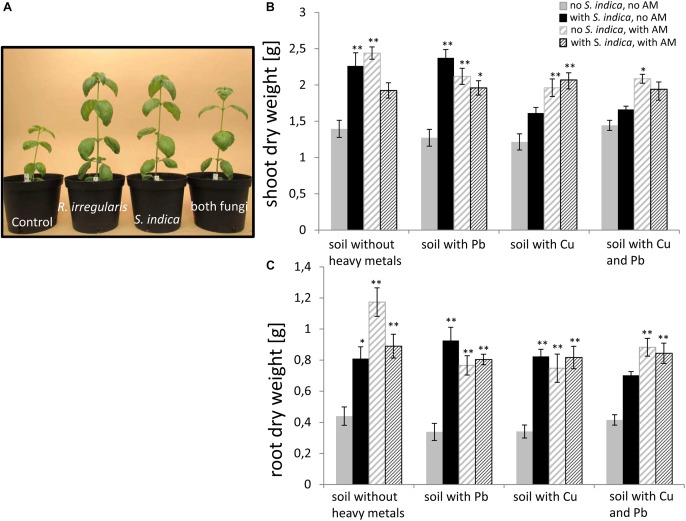
**(A)** Representative sweet basil plants inoculated with different fungi growing in uncontaminated soil. **(B)** Sweet basil shoot and **(C)** root dry weight after 5 weeks inoculation with the AM fungus *R. irregularis*, with *S. indica*, and with both fungi under different heavy metal treatments. Gray bars indicate non-inoculated sweet basil plants (control), black bars indicate plants inoculated only with *S. indica*, dashed gray bars indicate plants inoculated only with *R. irregularis*, and dashed black bars indicate plants inoculated with both fungi. Bars represent the mean out of nine individual plants with standard error. Asterisks indicate a significant difference compared to the respective non-inoculated control at the different heavy metal treatment according to Tukey’s HSD test (see Supplementary Table S2), ^∗^*p* ≤ 0.05, ^∗∗^*p* ≤ 0.001.

In order to test the impact of the two fungi and heavy metal treatments on sweet basil growth, different growth parameters were measured 5 weeks after inoculation. These parameters were the plant height, the number of leaves per plant, the shoot fresh weight (Supplementary Figures S1, S2), and the shoot and root dry weight (Figure 2). No obvious symptoms (Figure 2A) and no change in dry matter were visible on the plant parts of sweet basil growing on heavy metal contaminated soil (Figures 2B,C). Within our 144 plants we saw only 5 plants randomly distributed in the treatments with obvious disease symptoms (data not shown). An overall trend was visible that both fungi promoted an increase in biomasses (Figure 2). More precisely, sweet basil shoot dry weights were not changing with heavy metal treatment, but were significantly increased if roots were colonized with either *S. indica* or with *R. irregularis*. Interestingly, the increase was not significant when both fungi were present, in case of the combined Cu and Pb treatment and no heavy metal treatment. However, both fungi increased fresh weight significantly, although less than for the single fungal treatment (Supplementary Figure S2). Even under heavy metal treatment, shoot dry weight was significantly increased when the plants were mycorrhizal with *R. irregularis*. In contrast, *S. indica* conferred a growth promotion only in uncontaminated soil and soil contaminated with lead (Figure 2B). When both fungi were present in heavy metal contaminated soil, the effect resembled the mycorrhization effect.

Root dry weight showed similar effects (Figure 2C); except that *S. indica* treated plants had significantly higher root dry weight also when the soil was contaminated with copper. Also, shoot fresh weight, plant height and number of leaves showed similar responses to heavy metals and root colonization with a few minor exceptions (Supplementary Figures S1, S2).

### Phosphorus (P) Uptake

**FIGURE 3:**
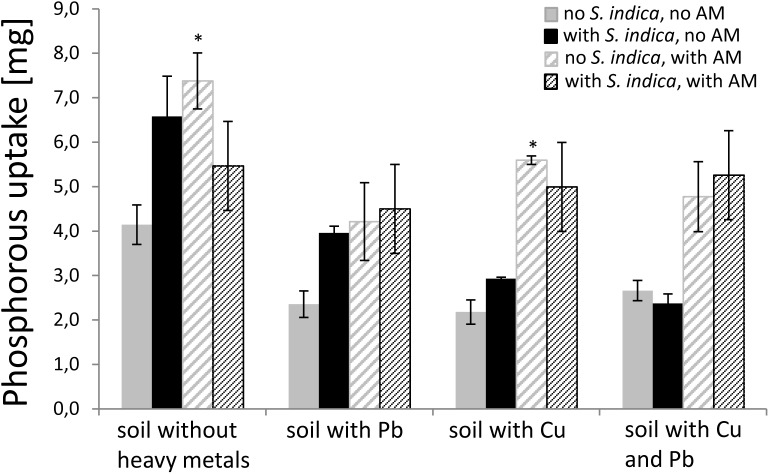
P uptake (P content × dry weight) in sweet basil shoots 5 weeks after inoculation with the AM fungus *R. irregularis*, with *S. indica*, and with both fungi under different heavy metal treatments. Bars are patterned as described for Figure 2. Asterisks indicate a significant difference compared to the respective non-inoculated control at the different heavy metal treatment according to Tukey’s HSD test (see Supplementary Table S3), ^∗^*p* ≤ 0.05

Since it is known that AMF increase the P uptake (P concentration [mg/g] × DW [g]) in plants, we measured the P concentration in the dried shoot material to confirm the mycorrhizal effect and to examine the effect of *S. indica* under the different heavy metal treatments. Mycorrhizal plants untreated with heavy metals showed a significant increase in P uptake (Figure 3). This effect was also visible when the soil was contaminated with copper but not when the soil was contaminated with lead or both heavy metals. Plants inoculated with *S. indica* showed a tendency to have a higher P uptake in uncontaminated soil and soil contaminated with lead or copper, respectively. When both fungi were present, the effect disappeared in non-contaminated soils, but resembled the mycorrhization effect, if heavy metals were present.

### Lead and Copper Concentration in Shoots and Roots

No lead was found in the plants growing on uncontaminated soil and in the soil treated with copper. Less than 0.3 mg/kg copper was found in the plant material harvested from uncontaminated soil and in the soil treated with lead (data not shown).

**FIGURE 4:**
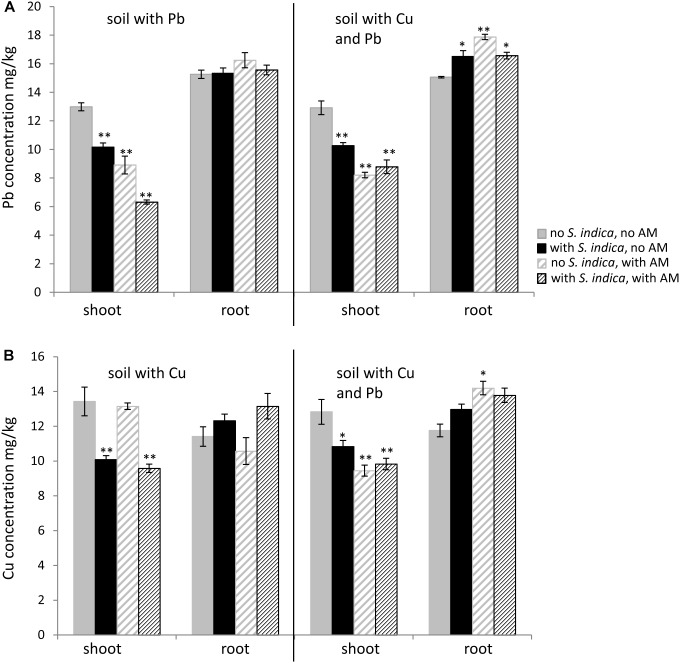
Lead (Pb) and copper (Cu) concentration in sweet basil roots and shoots 5 weeks after inoculation with the AM fungus *R. irregularis*, with *S. indica* and with both fungi under different heavy metal treatments. **(A)** Pb concentration in shoots and roots which grow on soil contaminated with Pb and both heavy metals, respectively. **(B)** Cu concentration in shoots and roots which grow on soil contaminated with Cu and both heavy metals, respectively. Bars are patterned as described for Figure 2 and represent the mean out of three plants with standard error. Asterisks indicate a significant difference compared to the respective non-inoculated control at the different heavy metal treatments according to Tukey’s HSD test (see Supplementary Tables S4, S5), ^∗^*p* ≤ 0.05, ^∗∗^*p* ≤ 0.001.

The lead concentration in sweet basil shoots decreased significantly when the plants were colonized with *R. irregularis* or *S. indica*, with the lowest amount being measured when both fungi were present (Figure 4A). The lead concentration in the shoot decreased from 12,98 mg/kg without fungal inoculation to 6,32mg/kg when the plants were inoculated with both fungi. No differences were found in the lead concentrations in the roots. When the soil was contaminated with both heavy metals, fungal inoculations had a similar effect in the shoots, however plants colonized with both fungi did not show a lower value compared to the mycorrhizal plants. In contrast to the roots growing on lead contaminated soil, the roots on soil contaminated with both heavy metals showed an increase in lead concentration when inoculated with the fungi (Figure 4A).

Interestingly the copper concentration in the shoots decreased only when the plants were inoculated with *S. indica*, not when they were only mycorrhizal (Figure 4B). In this case the copper concentration in the shoots remained stable at 13 mg/kg. Fungal treatments did not have any effect on the copper concentration in the roots when the soil was contaminated with copper. When both heavy metals were present in the soil, the copper concentration decreased in shoots for all fungal treatments (Figure 4B). The copper concentration in the roots increased significantly only when both heavy metals were present and when the roots were mycorrhizal.

### Essential Oils in Sweet Basil

**FIGURE 5:**
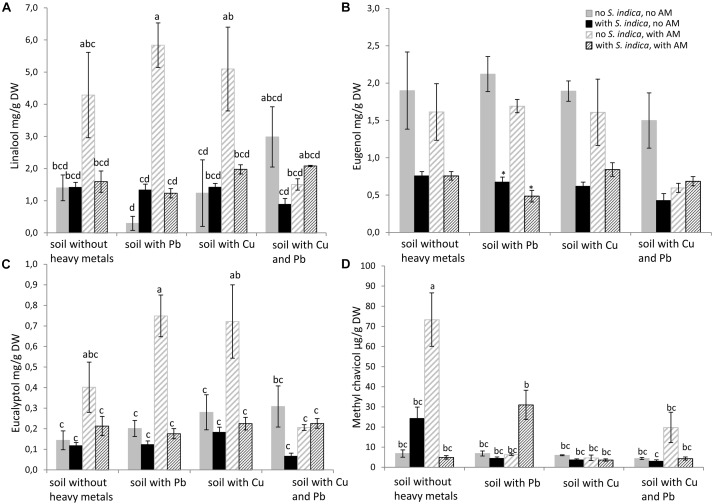
Essential oil concentration in sweet basil shoots 5 weeks after inoculation with the AM fungus *R. irregularis*, with *S. indica* and with both fungi under different heavy metal treatments. **(A)** Linalool concentration, **(B)** Eugenol concentration, **(C)** Eucalyptol concentration, **(D)** Methyl chavicol concentration. Bars are patterned as described for Figure 2 and represent the mean out of three plants with standard error. Letters indicate a significant difference at *p* ≤ 0.05 according to Tukey’s HSD test where all factors do interact. Asterisks indicate a significant difference compared to the respective non-inoculated control at the different heavy metal treatments according to Tukey’s HSD test (see Supplementary Tables S6, S7), ^∗^*p* ≤ 0.05.

Linalool, eugenol, eucalyptol and methyl chavicol were measured by GC in sweet basil shoots. Remarkably, the linalool concentration was increased only when the plants were mycorrhizal. Linalool levels increased even when the soil was contaminated with either lead or copper, but not when both heavy metals were present (Figure 5A). The eugenol concentration was impaired when the roots were colonized with *S. indica*, but this effect was only significant when the soil was contaminated with lead. *R. irregularis* seemed to have no effect on the eugenol concentration (Figure 5B). The effect of mycorrhization on eucalyptol resembled the effect on linalool. It increased when the plants grow in uncontaminated soil and in soil containing lead or copper. But eucalyptol did not increase in mycorrhizal plants when both heavy metals were present in the soil (Figure 5C). The methylchavicol concentration was dramatically increased when the roots were mycorrhizal. This effect was only visible in uncontaminated soil and not in the soils contaminated with heavy metals (Figure 5D).

## Discussion

### Fungal Colonization

The majority of vegetable and ornamental crops are host plants for AM fungi (AMF). Growth promotion of plants after inoculation with AMF is known for diverse species, including *O. basilicum* (Toussaint et al., 2008; Baum et al., 2015). The effect of AMF on *O. basilicum* is, however, species specific. Of the previously analyzed AMF *Funneliformis mosseae*, *Gigaspora margarita* and *Gigaspora rosea*, only *F. mosseae* induced significant increase in total biomass of *O. basilicum* plants (Copetta et al., 2006). Contributing to this literature, our study showed that sweet basil is a very good host for *R. irregularis* and hence the fungal inoculation might help producers to improve sweet basil growth and quality and support agricultural sustainability.

It is known that soil conditions and the presence of heavy metals negatively influence the interaction of beneficial fungi with plants (Yang et al., 2016). Prasad et al. (2011) found different mycorrhization levels in sweet basil due to the application of various heavy metals (Cr, Cd, Pb, and Ni) at different concentrations. Yet, lead had no influence on the mycorrhization level in sweet basil roots in our study, although we chose a high lead concentration. This might be due to the fact that *R. irregularis* seems to be less active in our experimental setup which leads to a reduced phosphorous uptake of the plant shown in Figure 3. In contrast, copper decreased the mycorrhization level to around 50%. Copper accumulation in the soil often originates from copper containing fungicides. These are used against pathogenic fungi of the aerial parts of the plant (Brunetto et al., 2016) which in turn might harm the microbial community in the soil.

Interestingly, the mycorrhization level increased with the simultaneous inoculation with *S. indica* in all treatments, except when lead was present in the soil (Figure 1). For tomato, Sartipnia et al. (2013) showed an increased activity of antioxidant enzymes after lead treatment. Such a defense response might influence colonization of beneficial fungi also in sweet basil. Additionally, Gill et al. (2016) reported a different colonization pattern of *S. indica* compared to AMF: They described a biphasic root colonization strategy with a biotrophic colonization pattern where an immune suppression was considered as a precondition for successful root colonization, although this is very host specific (Lahrmann et al., 2013; Gill et al., 2016). Hence we speculate that the *S. indica*-conferred immune suppression might enhance the colonization of AMF in our experiment. This is supported by the fact that wild lettuce inoculated with both mycorrhizal and endophytic fungi enjoyed more benefits than that with a single fungus in a polluted environment (Wazny et al., 2018). The mechanisms and degree of interaction and/or competition between *R. irregularis* and *S. indica* open new research opportunities for the future.

### Growth Parameters, Lead and Copper Concentration in Plant Matter and Phosphorous Uptake

#### Growth Parameters

Although not always significant, there was a general trend of increased growth of sweet basil in the presence of the used fungi (Figure 2 and Supplementary Figures S1, S2). This is in agreement with other studies showing that beneficial fungi improve plant growth also in disturbed and polluted sites (Christie et al., 2004; Prasad et al., 2011).

##### Lead

Even though both fungi improve growth in shoots and roots when the soil is contaminated with lead, the increase in growth is lower for *R. irregularis* treated plants compared to plants treated with *S. indica* (Figure 2). Yang et al. (2016) also discovered a decreased growth promotion with different lead concentrations on mycorrhizal *Robinia pseudoacacia*. Interestingly, in our experiment, lead had no influence on the growth promoting effect of *S. indica*. This is in agreement with observations that barley plants inoculated with *S. indica* are more resistant to lead than the non-colonized controls (Karimi et al., 2011). Depending on the host plant, *S. indica* might be more suited for plant benefits than AM fungi when the soil is contaminated with lead.

It has been proposed that AM fungi suppress salicylic acid (SA) signaling during later stages of colonization allowing a biotrophic interaction (Cameron et al., 2013). In contrast, the early biotrophic stage of *S. indica* colonization is followed by a programmed cell death-associated later stage (Lahrmann and Zuccaro, 2012). This later stage might be accompanied by SA accumulation, and SA-regulated genes are induced by *S. indica* under drought stress (Zhang et al., 2018a). This difference during late stages of colonization might explain the better performance of *S. indica*-inoculated plants under lead stress, since an alleviative effect of SA has been shown when wheat seedlings were treated with 200 mg/L lead (Song et al., 2012). Furthermore *S. indica* might be able to recruit jasmonic acid signaling to counterbalance the salicylic acid defense pathway in Arabidopsis (Jacobs et al., 2011). Therefore it might be interesting to measure the activity of oxidative stress enzymes if they are altered like Song et al. (2012) did for wheat. Both fungi are important players when plants deal with oxidative stress (Schützendübel and Polle, 2002; Nath et al., 2016), but if and how this happens in our experimental setup needs further investigations.

##### Copper

*S. indica* was not able to significantly improve growth of the shoots of sweet basil when copper was present in the soil. For barley, Karimi et al. (2011) showed that the growth promoting effect was dependent on copper levels in the soil. Sweet basil inoculated with *R. irregularis* and grown in copper contaminated soil still showed a significant growth promotion on the shoots and the roots in our experiment (Figure 2). Plant biomass, root density, chlorophyll, carotenoid, and protein content significantly increased in *Glycine max.* with the inoculation of *Penicillium funiculosum* LHL06 to counteract copper stress by secreting bioactive gibberellins to bind the metal into stable complexes (Khan and Lee, 2013). In contrast it is known that gibberellins have a negative effect on arbuscule formation in pea and tomato (Foo et al., 2013; Martín-Rodríguez et al., 2016). Copper has no influence on sweet basil shoot dry weight in the presence of *S. indica*. In contrast to AM fungi, *S. indica* colonizes plant roots mainly extracellularly. We therefore hypothesize that copper, which can act as a fungicide, harms the fungus and thus limits its growth promoting effects to the shoot. This still needs to be verified by, for example, determining the colonization level of *S. indica* under different heavy metal stresses.

#### Heavy Metal Concentration

AMF colonizing plant roots were shown to reduce the uptake of heavy metals into plant cells (Hildebrandt et al., 2007). This can take place by changing the soil structure and chemical stabilization of metals in the soil (Gaur and Adholeya, 2004), decreasing the risk of toxicity to plants (Gonzalez-Chavez et al., 2009). In contrast, Dietterich et al. (2017) found no effects of AMF colonization on plant metal uptake, whereas a promotion of heavy metal uptake with AMF and *S. indica* was described for sunflower (Shahabivand et al., 2017; Zhang et al., 2018b). Furthermore, Park et al. (2016) showed that peat moss supports mobility and bioavailability of lead and copper. This indicates that the effect of AMF to heavy metal toxicity in plants is highly dependent on the plant host species, the fungal isolate and the soil structure.

##### Lead

For plants, it is known that most of the lead taken up remains in the roots (Kumar et al., 1995). In our experiment we saw that the lead concentration significantly decreased in the shoots but not in the roots inoculated with AMF (Figure 4). Different effects were described in the shoots for mycorrhizal plants growing on contaminated soil. Rydlová and Vosátka (2003) observed that mycorrhizal *Agrostis capillaris* took up more lead, whereas Yang et al. (2016) showed that different mycorrhizal plant species took up less lead when contamination is very high. For *Origanum majorana* it was shown that the lead content was reduced in shoots and roots when inoculated with the AM fungi *F. mosseae*, but lead content increased when infected with *Claroideoglomus claroideum* (Hristozkova et al., 2015). Surprisingly, in our experiment the lead concentration in the inoculated sweet basil roots increased, when both heavy metals were present in the soil. This effect was more significant in mycorrhizal roots than in roots inoculated with *S. indica*. The ability of *S. indica* to lower the pH in the surrounding media is also known for the rhizosphere in general (Lin et al., 2004; Ngwene et al., 2016). A decrease of the pH might lead to a higher absorption of lead, as shown for *Oryza sativa* (Lin et al., 2004). In contrast, the lead availability is reduced in the *Pelargonium hortorum* rhizosphere, when other heavy metals with antagonistic effects, such as copper, are present (Orroño et al., 2012). Since there are no results in the literature on the interaction between mycorrhizal fungi and *S. indica*, we can only speculate that in our experiments both fungi have an additive effect to decrease the uptake of lead by the shoots. Further investigations are needed to verify this conjecture.

##### Copper

Given our setup, the copper concentration in the sweet basil shoots decreased significantly when the roots are inoculated with *S. indica* but not when they are mycorrhizal. The exact mechanisms responsible for this difference are unknown at present. One explanation could be that some fungi are able to use avoidance mechanisms or compartmentalization strategies to detoxify metal cations (Meharg, 2003). The mycelia architecture of *R. irregularis* alters considerably under copper concentrations, at amounts lethal to other organisms (Tamayo et al., 2014; Ruscitti et al., 2017). Although Tamayo et al. (2014) revealed the presence of 30 metal transporters for copper, iron (Fe), and zinc (Zn) in *R. irregularis*, the exact function and purpose of these transporters in the *R. irregularis* membranes are unknown. Our results indicate that a comparison between the copper transporters in *R. irregularis* and *S. indica* might be helpful to explain the different effects of the two fungi on copper uptake by sweet basil.

#### Phosphorous Uptake

We showed that the mycorrhization effect on sweet basil roots was impaired by lead and not by copper (Figure 3). The ability of *S. indica* to support plant nutrition is still under investigation. Ngwene et al. (2016) found that *S. indica* is able to solubilize phosphate from inorganic phosphorus by lowering the pH, rather than an enzymatic effect. Our results show that *S. indica* impairs the mycorrhizal effect in the uncontaminated soil, which is in contradiction to our finding that plants inoculated with *S. indica* had a higher mycorrhizal colonization. Therefore this increase in mycorrhizal colonization may not have a biological relevance.

### Essential Oil Concentrations

Many studies investigate the composition and biosynthesis of essential oils in sweet basil. We observed a significant interaction between the fungi and the heavy metal treatments on the linalool, eucalyptol and methyl chavicol concentrations (Figure 5). A clear increase in linalool and eucalyptol concentration was visible when the roots were inoculated with *R. irregularis* irrespective of the heavy metal treatment. Even though, the mechanisms are not very well studied, we suspect that the increase of certain essential oils in sweet basil inoculated with AMF might be due to increased nutrient uptake. However, Copetta et al. (2006) and Khaosaad et al. (2006) suggested that an altered P uptake is not be responsible for variations observed in the essential oil production in plants belonging to the *Lamiaceae* family. A possible explanation might be that methyl chavicol and eugenol belong to the phenylpropenes which have a different biosynthesis pathway than the terpenes linalool and eucalyptol. Prasad et al. (2011) found an increase in linalool content in the presence of lead, but methyl chavicol levels were unaffected in their study. Even though they examined *O. basilicum* in a pot experiment, differences in their experimental conditions such as soil structure, plant age and heavy metal content prevent direct comparison to our results. Moreover, Hristozkova et al. (2015) found that different isolates of *F. mosseae* and *C. claroideum* have changing effects on the composition of essential oils in *O. majorana*, supporting our hypothesis that the AM fungus is the active component affecting essential oil biosynthesis. Research on the ability of fungi to produce bioactive fungal metabolites is of high interest for the production of organic crops (Rai et al., 2014).

In contrast to our results, two studies showed that *S. indica* colonized herbs, such as fennel and *Thymus vulgaris*, harbor higher amounts of essential oil (Dolatabadi et al., 2011a,b). As we mentioned above, the effects of *S. indica* are very host dependent. Our results indicate that *S. indica* might not be suitable to increase the essential oil content in sweet basil. In fact, *S. indica* decreased the eugenol concentration.

## Perspectives and Conclusion

A tremendous amount of literature is available on the role of phytohormones related to plant-microbe interactions. Results range from how mycorrhizal fungi develop and colonize plant roots – which in some cases are mutated in specific phytohormone pathways – to how this has an impact on plant fitness. The more research was done the more complex the situation became (Pieterse et al., 2014). There is also ample research on the effects of plant phytohormones to heavy metal stress (Ben Massoud et al., 2018). Our experimental setup has the potential to shed light on many of these processes if phytohormone levels and specific gene expression levels measurements are included. Using the resulting data checking a putative connection to the essential oil production of sweet basil seems worthwhile, as it is known that these secondary metabolites serve as defense and signal compounds. Furthermore, their biosynthesis is also regulated by phytohormones.

The industrially used herb *O. basilicum* represents a suitable host for both the beneficial fungi *R. irregularis* and *S. indica.* Both fungi have the ability to increase shoot and root weights after inoculation. Particularly, these increases were also visible in the presence of heavy metals in the soil. Linalool and Eucalyptol concentrations were dramatically changed in the presence of *R. irregularis* but not in the presence of *S. indica*. Furthermore both fungi protected sweet basil shoots from lead uptake, but only *S. indica* was able to protect the plants from copper uptake. For this reason, the use of beneficial fungi might be a helpful tool to lower heavy metal concentration in final agricultural products and to improve essential oil contents.

## Author Contributions

MS, AA, and PF initiated the research. MS and PF designed the experiments. MS performed the experiments. IC directed the molecular work and supervised the execution of the experiments. EP and LW measured the essential oils. MS, PF, and IC analyzed the data. IC, MS, PF, and LW wrote the manuscript.

## Conflict of Interest Statement

The authors declare that the research was conducted in the absence of any commercial or financial relationships that could be construed as a potential conflict of interest.
