# Supplementary material for: Beneficial Root Endophytic Fungi Increase Growth and Quality Parameters of Sweet Basil in Heavy Metal Contaminated Soil
Source: Front Plant Sci. 2018 Nov 27;9:1726. doi: 10.3389/fpls.2018.01726 (PMC6277477; doi:10.3389/fpls.2018.01726)
Supplement: Supplementary file 1 [file Table_1.DOCX]

Table S1: Results of a three way ANOVA associated with Figure 1B. s: significant impact or interaction.

| Factor | Degr. of Freedom | *F* | *p* | Mycorrhization |
| --- | --- | --- | --- | --- |
| Heavy metal | 3 | 70,39 | 0,000 | s |
| *S. indica* | 1 | 21,24 | 0,000 | s |
| *R. irregularis* | 1 | 28038,48 | 0,000 | s |
| Heavy metal * *S. indica* | 3 | 5,48 | 0,003 | s |
| Heavy metal * *R. irregularis* | 3 | 70,39 | 0,000 | s |
| *S. indica* * *R. irregularis* | 1 | 21,24 | 0,000 | s |
| Heavy metal * *S. indica* * *R. irregularis* | 3 | 5,48 | 0,003 | s |
