# Supplementary material for: Beneficial Root Endophytic Fungi Increase Growth and Quality Parameters of Sweet Basil in Heavy Metal Contaminated Soil
Source: Front Plant Sci. 2018 Nov 27;9:1726. doi: 10.3389/fpls.2018.01726 (PMC6277477; doi:10.3389/fpls.2018.01726)
Supplement: Supplementary file 2 [file Table_2.DOCX]

Table S2: Results of a four way ANOVA (*p* = 0.05; *n* = 9) associated with Figure 2B and C for shoot dry weight (SDW) and root dry weight (RDW). s: significant impact or interaction, ns: no significant impact or interaction. Degrees of Freedom in all cases: 1.

| Factor | *F* (SDW) | *p* (SDW) | SDW | *F* (RDW) | *p* (RDW) | RDW |
| --- | --- | --- | --- | --- | --- | --- |
| Pb | 0,003 | 0,957 | ns | 2,024 | 0,157 | ns |
| Cu | 15,790 | 0,000 | s | 5,040 | 0,026 | s |
| *S. indica* | 17,966 | 0,000 | s | 34,942 | 0,000 | s |
| *R. irregularis* | 54,565 | 0,000 | s | 69,495 | 0,000 | s |
| Pb * Cu | 1,662 | 0,199 | ns | 5,394 | 0,021 | s |
| Pb * *S. indica* | 0,132 | 0,717 | ns | 0,857 | 0,356 | ns |
| Cu * *S. indica* | 2,631 | 0,107 | ns | 0,116 | 0,734 | ns |
| Pb * *R. irregularis* | 1,574 | 0,211 | ns | 1,370 | 0,244 | ns |
| Cu * *R. irregularis* | 5,014 | 0,027 | s | 0,186 | 0,666 | ns |
| *S. indica* * *R. irregularis* | 55,692 | 0,000 | s | 57,935 | 0,000 | s |
| Pb * Cu * *S. indica* | 5,329 | 0,022 | s | 10,888 | 0,001 | s |
| Pb * Cu * *R. irregularis* | 0,000 | 0,986 | ns | 7,895 | 0,005 | s |
| Pb * *S. indica* * *R. irregularis* | 0,013 | 0,909 | ns | 0,565 | 0,453 | ns |
| Cu * *S. indica* * *R. irregularis* | 20,315 | 0,000 | s | 3,279 | 0,072 | ns |
| Pb * Cu * *S. indica* * *R. irregularis* | 0,193 | 0,661 | ns | 0,003 | 0,954 | ns |
