# Supplementary material for: Beneficial Root Endophytic Fungi Increase Growth and Quality Parameters of Sweet Basil in Heavy Metal Contaminated Soil
Source: Front Plant Sci. 2018 Nov 27;9:1726. doi: 10.3389/fpls.2018.01726 (PMC6277477; doi:10.3389/fpls.2018.01726)
Supplement: Supplementary file 3 [file Table_3.DOCX]

Table S3: Results of a four way ANOVA (*p* = 0.05; *n* = 3) associated with Figure 3. s: significant impact or interaction, ns: no significant impact or interaction. Degrees of Freedom in all cases: 1.

| Factor | *F* | *p* | P uptake |
| --- | --- | --- | --- |
| Pb | 17,317 | 0,000 | s |
| Cu | 12,612 | 0,001 | s |
| *S. indica* | 1,563 | 0,220 | ns |
| *R. irregularis* | 46,279 | 0,000 | s |
| Pb * Cu | 12,874 | 0,001 | s |
| Pb * *S. indica* | 0,410 | 0,526 | ns |
| Cu * *S. indica* | 0,877 | 0,356 | ns |
| Pb * *R. irregularis* | 0,009 | 0,924 | ns |
| Cu * *R. irregularis* | 7,295 | 0,010 | s |
| *S. indica* * *R. irregularis* | 8,019 | 0,008 | s |
| Pb * Cu * *S. indica* | 0,356 | 0,554 | ns |
| Pb * Cu * *R. irregularis* | 0,118 | 0,733 | ns |
| Pb * *S. indica* * *R. irregularis* | 5,454 | 0,026 | s |
| Cu * *S. indica* * *R. irregularis* | 5,318 | 0,028 | s |
| Pb * Cu * *S. indica* * *R. irregularis* | 0,167 | 0,685 | ns |
