# Supplementary material for: Beneficial Root Endophytic Fungi Increase Growth and Quality Parameters of Sweet Basil in Heavy Metal Contaminated Soil
Source: Front Plant Sci. 2018 Nov 27;9:1726. doi: 10.3389/fpls.2018.01726 (PMC6277477; doi:10.3389/fpls.2018.01726)
Supplement: Supplementary file 4 [file Table_4.DOCX]

Table S4: Results of a four way ANOVA (*p* = 0.05; *n* = 3) associated with Figure 4A. s: significant impact or interaction, ns: no significant impact or interaction. Degrees of Freedom in all cases: 1.

| Factor | *F* | *p* | Pb shoot content | *F* | *p* | Pb root content |
| --- | --- | --- | --- | --- | --- | --- |
| Pb | 4186,637 | 0,000 | s | 14086,40 | 0,000 | s |
| Cu | 2,223 | 0,145 | ns | 11,16 | 0,002 | s |
| *S. indica* | 37,789 | 0,000 | s | 0,19 | 0,669 | ns |
| *R. irregularis* | 134,941 | 0,000 | s | 14,32 | 0,000 | s |
| Pb * Cu | 2,223 | 0,145 | ns | 11,16 | 0,002 | s |
| Pb * *S. indica* | 37,789 | 0,000 | s | 0,19 | 0,669 | ns |
| Cu * *S. indica* | 7,614 | 0,009 | s | 0,48 | 0,491 | ns |
| Pb * *R. irregularis* | 134,941 | 0,000 | s | 14,32 | 0,000 | s |
| Cu * *R. irregularis* | 2,015 | 0,165 | ns | 2,35 | 0,134 | ns |
| *S. indica* * *R. irregularis* | 8,075 | 0,007 | s | 10,55 | 0,002 | s |
| Pb * Cu * *S. indica* | 7,614 | 0,009 | s | 0,48 | 0,491 | ns |
| Pb * Cu * *R. irregularis* | 2,015 | 0,165 | ns | 2,35 | 0,134 | ns |
| Pb * *S. indica* * *R. irregularis* | 8,075 | 0,007 | s | 10,55 | 0,002 | s |
| Cu * *S. indica* * *R. irregularis* | 6,092 | 0,019 | s | 3,42 | 0,737 | ns |
| Pb * Cu * *S. indica* * *R. irregularis* | 6,092 | 0,019 | s | 3,42 | 0,737 | ns |
