# Supplementary material for: Beneficial Root Endophytic Fungi Increase Growth and Quality Parameters of Sweet Basil in Heavy Metal Contaminated Soil
Source: Front Plant Sci. 2018 Nov 27;9:1726. doi: 10.3389/fpls.2018.01726 (PMC6277477; doi:10.3389/fpls.2018.01726)
Supplement: Supplementary file 5 [file Table_5.DOCX]

Table S5: Results of a four way ANOVA (*p* = 0.05; *n* = 3) associated with Figure 4B. s: significant impact or interaction, ns: no significant impact or interaction. Degrees of Freedom in all cases: 1.

| Factor | *F* | *p* | Cu shoot content | *F* | *p* | Cu root content |
| --- | --- | --- | --- | --- | --- | --- |
| Pb | 5,424 | 0,026 | s | 7,605 | 0,009 | s |
| Cu | 3322,732 | 0,000 | s | 3322,686 | 0,000 | s |
| *S. indica* | 32,042 | 0,000 | s | 6,347 | 0,016 | s |
| *R. irregularis* | 10,731 | 0,002 | s | 3,540 | 0,069 | ns |
| Pb * Cu | 4,116 | 0,505 | ns | 11,485 | 0,001 | s |
| Pb * *S. indica* | 13,101 | 0,001 | s | 2,380 | 0,132 | ns |
| Cu * *S. indica* | 31,349 | 0,000 | s | 6,227 | 0,017 | s |
| Pb * *R. irregularis* | 5,631 | 0,023 | s | 4,275 | 0,046 | s |
| Cu * *R. irregularis* | 12,456 | 0,001 | s | 3,699 | 0,063 | ns |
| *S. indica* * *R. irregularis* | 2,039 | 0,163 | ns | 0,003 | 0,957 | ns |
| Pb * Cu * *S. indica* | 11,419 | 0,001 | s | 2,580 | 0,118 | ns |
| Pb * Cu * *R. irregularis* | 5,711 | 0,022 | s | 3,023 | 0,091 | ns |
| Pb * *S. indica* * *R. irregularis* | 3,387 | 0,075 | ns | 3,812 | 0,059 | ns |
| Cu * *S. indica* * *R. irregularis* | 2,004 | 0,166 | ns | 0,000 | 1,000 | ns |
| Pb * Cu * *S. indica* * *R. irregularis* | 2,566 | 0,119 | ns | 3,612 | 0,066 | ns |
