# Supplementary material for: Beneficial Root Endophytic Fungi Increase Growth and Quality Parameters of Sweet Basil in Heavy Metal Contaminated Soil
Source: Front Plant Sci. 2018 Nov 27;9:1726. doi: 10.3389/fpls.2018.01726 (PMC6277477; doi:10.3389/fpls.2018.01726)
Supplement: Supplementary file 6 [file Table_6.DOCX]

Table S6: Results of a four-way ANOVA (*p* = 0.05; *n* = 3) associated with Figure 5A and B. s: significant impact or interaction, ns: no significant impact or interaction. Degrees of Freedom in all cases: 1.

| Factor | *F* | *p* | Linalool | *F* | *p* | Eugenol |
| --- | --- | --- | --- | --- | --- | --- |
| Pb | 0,601 | 0,443 | ns | 2,820 | 0,102 | ns |
| Cu | 0,005 | 0,942 | ns | 2,883 | 0,099 | ns |
| *S. indica* | 13,473 | 0,000 | s | 50,748 | 0,000 | s |
| *R. irregularis* | 18,761 | 0,000 | s | 2,222 | 0,145 | ns |
| Pb * Cu | 0,609 | 0,440 | ns | 2,482 | 0,124 | ns |
| Pb * *S. indica* | 0,033 | 0,856 | ns | 0,142 | 0,708 | ns |
| Cu * *S. indica* | 0,374 | 0,545 | ns | 2,280 | 0,140 | ns |
| Pb * *R. irregularis* | 0,639 | 0,429 | ns | 0,722 | 0,401 | ns |
| Cu * *R. irregularis* | 2,250 | 0,143 | ns | 0,034 | 0,853 | ns |
| *S. indica* * *R. irregularis* | 9,468 | 0,004 | s | 4,152 | 0,049 | s |
| Pb * Cu * *S. indica* | 0,625 | 0,434 | ns | 2,529 | 0,121 | ns |
| Pb * Cu * *R. irregularis* | 5,883 | 0,021 | s | 0,055 | 0,814 | ns |
| Pb * *S. indica* * *R. irregularis* | 1,081 | 0,306 | ns | 0,3167 | 0,577 | ns |
| Cu * *S. indica* * *R. irregularis* | 7,008 | 0,012 | s | 1,1214 | 0,297 | ns |
| Pb * Cu * *S. indica* * *R. irregularis* | 9,331 | 0,004 | s | 0,418 | 0,522 | ns |
