# Supplementary material for: Beneficial Root Endophytic Fungi Increase Growth and Quality Parameters of Sweet Basil in Heavy Metal Contaminated Soil
Source: Front Plant Sci. 2018 Nov 27;9:1726. doi: 10.3389/fpls.2018.01726 (PMC6277477; doi:10.3389/fpls.2018.01726)
Supplement: Supplementary file 7 [file Table_7.DOCX]

Table S7: Results of a four-way ANOVA (*p* = 0.05; *n* = 3) associated with Figure 5C and D. s: significant impact or interaction, ns: no significant impact or interaction. Degrees of Freedom in all cases: 1.

| Factor | *F* | *p* | Eucalyptol | *F* | *p* | Methyl chavicol |
| --- | --- | --- | --- | --- | --- | --- |
| Pb | 0,470 | 0,497 | ns | 5,112 | 0,030 | s |
| Cu | 0,073 | 0,787 | ns | 26,972 | 0,000 | s |
| *S. indica* | 25,308 | 0,000 | s | 5,468 | 0,025 | s |
| *R. irregularis* | 20,000 | 0,000 | s | 18,040 | 0,000 | s |
| Pb * Cu | 8,493 | 0,006 | s | 12,613 | 0,001 | s |
| Pb * *S. indica* | 0,037 | 0,847 | ns | 8,200 | 0,007 | s |
| Cu * *S. indica* | 0,022 | 0,882 | ns | 0,166 | 0,686 | ns |
| Pb * *R. irregularis* | 0,292 | 0,592 | ns | 0,019 | 0,888 | ns |
| Cu * *R. irregularis* | 1,534 | 0,224 | ns | 7,619 | 0,009 | s |
| *S. indica* * *R. irregularis* | 5,644 | 0,023 | s | 11,723 | 0,001 | s |
| Pb * Cu * *S. indica* | 5,844 | 0,021 | s | 17,173 | 0,000 | s |
| Pb * Cu * *R. irregularis* | 4,059 | 0,052 | ns | 3,517 | 0,069 | ns |
| Pb * *S. indica* * *R. irregularis* | 0,971 | 0,0331 | ns | 21,873 | 0,000 | s |
| Cu * *S. indica* * *R. irregularis* | 2,420 | 0,129 | ns | 4,827 | 0,035 | s |
| Pb * Cu * *S. indica* * *R. irregularis* | 8,821 | 0,005 | s | 37,663 | 0,000 | s |
