# Supplementary material for: Beneficial Root Endophytic Fungi Increase Growth and Quality Parameters of Sweet Basil in Heavy Metal Contaminated Soil
Source: Front Plant Sci. 2018 Nov 27;9:1726. doi: 10.3389/fpls.2018.01726 (PMC6277477; doi:10.3389/fpls.2018.01726)
Supplement: Supplementary file 8 [file Table_8.DOCX]

Table S8: Results of a four-way ANOVA (*p* = 0.05; *n* = 3) associated with Figure S1. s: significant impact or interaction, ns: no significant impact or interaction. Degrees of Freedom in all cases: 1.

| Factor | *F* | *p* | Plant height | *F* | *p* | Number of leaves |
| --- | --- | --- | --- | --- | --- | --- |
| Pb | 20,193 | 0,000 | s | 34,047 | 0,000 | s |
| Cu | 37,647 | 0,000 | s | 26,881 | 0,000 | s |
| *S. indica* | 31,952 | 0,000 | s | 41,592 | 0,000 | s |
| *R. irregularis* | 91,945 | 0,000 | s | 110,921 | 0,000 | s |
| Pb * Cu | 0,300 | 0,585 | ns | 4,467 | 0,036 | s |
| Pb * *S. indica* | 1,985 | 0,161 | ns | 0,144 | 0,705 | ns |
| Cu * *S. indica* | 6,681 | 0,010 | s | 17,414 | 0,000 | s |
| Pb * *R. irregularis* | 0,760 | 0,385 | ns | 0,447 | 0,505 | ns |
| Cu * *R. irregularis* | 4,705 | 0,031 | s | 13,984 | 0,000 | s |
| *S. indica* * *R. irregularis* | 52,524 | 0,000 | s | 33,627 | 0,000 | s |
| Pb * Cu * *S. indica* | 1,359 | 0,245 | ns | 1,465 | 0,228 | ns |
| Pb * Cu * *R. irregularis* | 0,101 | 0,751 | ns | 0,173 | 0,678 | ns |
| Pb * *S. indica* * *R. irregularis* | 1,910 | 0,169 | ns | 1,836 | 0,177 | ns |
| Cu * *S. indica* * *R. irregularis* | 11,900 | 0,000 | s | 2,037 | 0,155 | ns |
| Pb * Cu * *S. indica* * *R. irregularis* | 0,630 | 0,428 | ns | 0,496 | 0,482 | ns |
