# Supplementary material for: Beneficial Root Endophytic Fungi Increase Growth and Quality Parameters of Sweet Basil in Heavy Metal Contaminated Soil
Source: Front Plant Sci. 2018 Nov 27;9:1726. doi: 10.3389/fpls.2018.01726 (PMC6277477; doi:10.3389/fpls.2018.01726)
Supplement: Supplementary file 9 [file Table_9.DOCX]

Table S9: Results of a four-way ANOVA (*p* = 0.05; *n* = 3) associated with Figure S2. s: significant impact or interaction, ns: no significant impact or interaction. Degrees of Freedom in all cases 1.

| Factor | *F* | *p* | Shoot fresh weight |
| --- | --- | --- | --- |
| Pb | 11,746 | 0,001 | s |
| Cu | 22,229 | 0,000 | s |
| *S. indica* | 29,218 | 0,000 | s |
| *R. irregularis* | 86,653 | 0,000 | s |
| Pb * Cu | 5,795 | 0,017 | s |
| Pb * *S. indica* | 0,138 | 0,710 | ns |
| Cu * *S. indica* | 6,090 | 0,014 | s |
| Pb * *R. irregularis* | 0,147 | 0,702 | ns |
| Cu * *R. irregularis* | 8,680 | 0,003 | s |
| *S. indica* * *R. irregularis* | 63,647 | 0,000 | s |
| Pb * Cu * *S. indica* | 6,494 | 0,012 | s |
| Pb * Cu * *R. irregularis* | 0,053 | 0,817 | ns |
| Pb * *S. indica* * *R. irregularis* | 5,130 | 0,025 | s |
| Cu * *S. indica* * *R. irregularis* | 16,896 | 0,000 | s |
| Pb * Cu * *S. indica* * *R. irregularis* | 0,250 | 0,617 | ns |
