# Supplementary material for: Beneficial Root Endophytic Fungi Increase Growth and Quality Parameters of Sweet Basil in Heavy Metal Contaminated Soil
Source: Front Plant Sci. 2018 Nov 27;9:1726. doi: 10.3389/fpls.2018.01726 (PMC6277477; doi:10.3389/fpls.2018.01726)
Supplement: Supplementary file 10 [file Image_1.PDF]

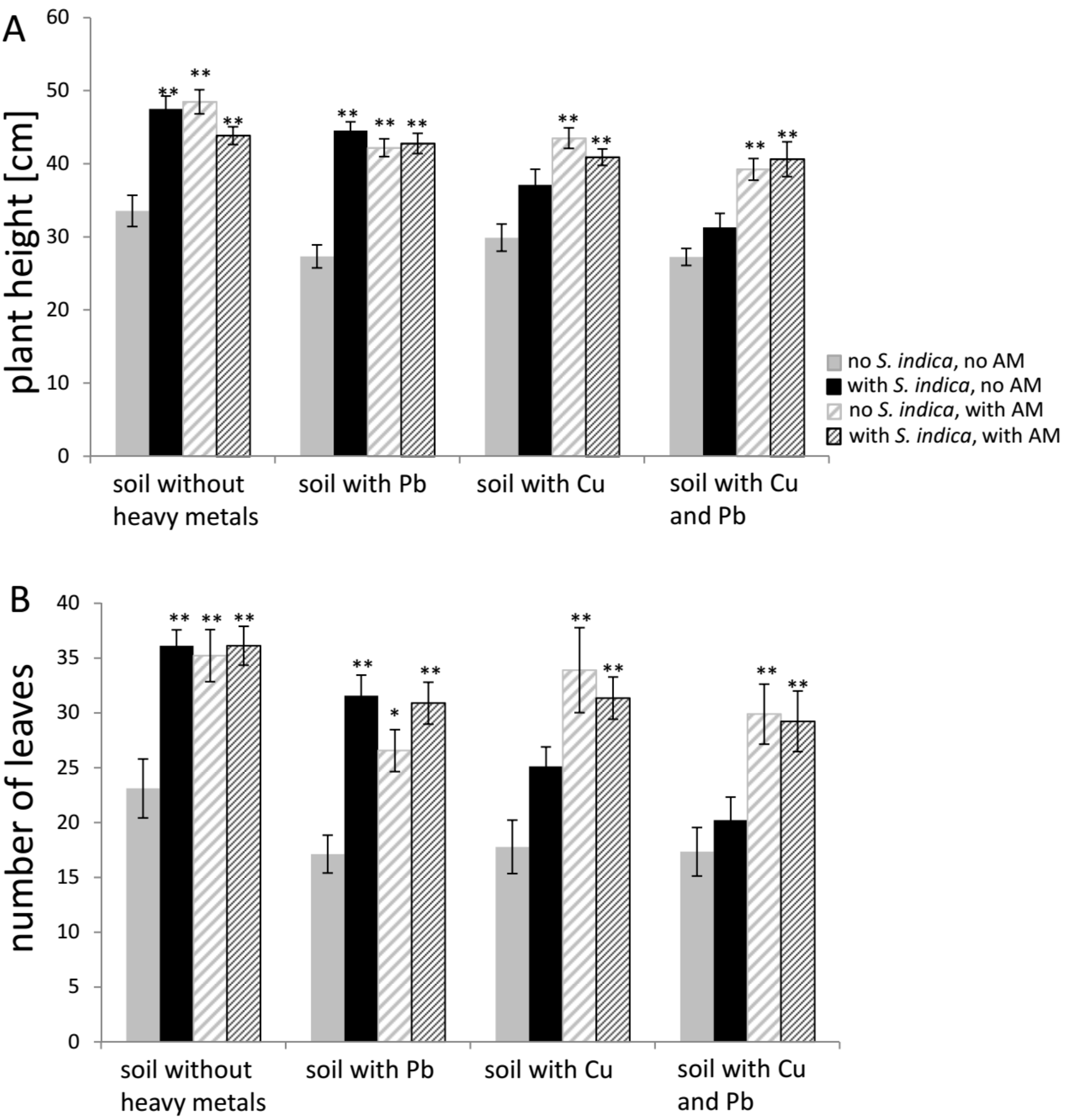

Figure S1: Plant height (A) and number of leaves (B) five weeks after inoculation with the AM fungus *R. irregularis*, with *S. indica* and with both fungi under different heavy metal treatments. Grey bars indicate non-inoculated sweet basil plants (control), black bars indicate plants inoculated inly with *S. indica*, dashed grey bars indicate plants inoculated only with *R. irregularis* and, dashed black bars indicate plants inoculated with both fungi. Bars represent the mean out of nine plants with standard error. Asterisks indicate a significant difference compared to the respective non-inoculated control at the different heavy metal treatment according to Tukey's HSD test, ** $p \leq 0.001$, * $p \leq 0.05$.
