# Supplementary material for: Beneficial Root Endophytic Fungi Increase Growth and Quality Parameters of Sweet Basil in Heavy Metal Contaminated Soil
Source: Front Plant Sci. 2018 Nov 27;9:1726. doi: 10.3389/fpls.2018.01726 (PMC6277477; doi:10.3389/fpls.2018.01726)
Supplement: Supplementary file 11 [file Image_2.PDF]

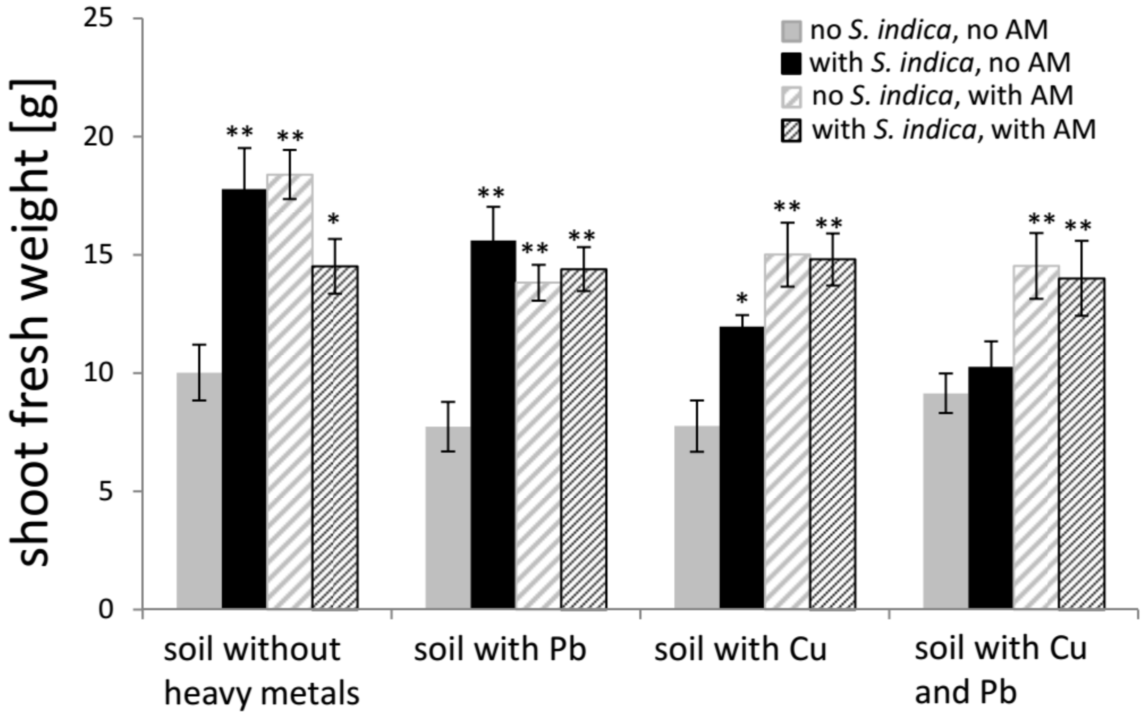

Figure S2: Sweet basil shoot fresh weight five weeks after inoculation with the AM fungus *R. irregularis*, with *S. indica* and with both fungi under different heavy metal treatments. Bars are patterned as described for Fig. S1 and represent the mean out of sine plants with standard error. Asterisks indicate a significant difference compared to the respective non-inoculated control at the different heavy metal treatment according to Tukey’s HSD test, * $p \leq 0.05$, ** $p \leq 0.001$.
